# Scratching the Surface: Bacterial Cell Envelopes at the Nanoscale

**DOI:** 10.1128/mBio.03020-19

**Published:** 2020-02-25

**Authors:** Albertus Viljoen, Simon J. Foster, Georg E. Fantner, Jamie K. Hobbs, Yves F. Dufrêne

**Affiliations:** a Louvain Institute of Biomolecular Science and Technology, UCLouvain, Louvain-la-Neuve, Belgium; b Krebs Institute, University of Sheffield, Sheffield, South Yorkshire, United Kingdom; c Laboratory for Bio- and Nano-Instrumentation, Swiss Federal Institute of Technology Lausanne (EPFL), Lausanne, Switzerland; d Department of Physics and Astronomy, University of Sheffield, Sheffield, South Yorkshire, United Kingdom; e Walloon Excellence in Life Sciences and Biotechnology (WELBIO), Wavre, Belgium; University of Texas Health Science Center at Houston

**Keywords:** bacterial envelopes, ultrastructure, drugs, imaging, atomic force microscopy, assembly, microscopy

## Abstract

The bacterial cell envelope is essential for viability, the environmental gatekeeper and first line of defense against external stresses. For most bacteria, the envelope biosynthesis is also the site of action of some of the most important groups of antibiotics. It is a complex, often multicomponent structure, able to withstand the internally generated turgor pressure. Thus, elucidating the architecture and dynamics of the cell envelope is important, to unravel not only the complexities of cell morphology and maintenance of integrity but also how interventions such as antibiotics lead to death. To address these questions requires the capacity to visualize the cell envelope *in situ* via high-spatial resolution approaches. In recent years, atomic force microscopy (AFM) has brought novel molecular insights into the assembly, dynamics, and functions of bacterial cell envelopes. The ultrafine resolution and physical sensitivity of the technique have revealed a wealth of ultrastructural features that are invisible to traditional optical microscopy techniques or imperceptible in their true physiological state by electron microscopy. Here, we discuss recent progress in our use of AFM imaging for understanding the architecture and dynamics of the bacterial envelope. We survey recent studies that demonstrate the power of the technique to observe isolated membranes and live cells at (sub)nanometer resolution and under physiological conditions and to track *in vitro* structural dynamics in response to growth or to drugs.

## Abstract (precis)

The bacterial cell envelope is essential for viability, the environmental gatekeeper and first line of defense against external stresses. For most bacteria, the envelope biosynthesis is also the site of action of some of the most important groups of antibiotics. It is a complex, often multicomponent structure, able to withstand the internally generated turgor pressure. Thus, elucidating the architecture and dynamics of the cell envelope is important, to unravel not only the complexities of cell morphology and maintenance of integrity but also how interventions such as antibiotics lead to death.

## THE COMPLEX ARCHITECTURE OF BACTERIAL ENVELOPES

The envelopes surrounding bacterial cytosols are structurally and functionally complex and diverse, allowing them to cope with the equally diverse, volatile, and often hostile environments that they inhabit (1–3). Cell envelopes confer specialized functions that are important for bacterial physiology and that often are crucial to symbiosis, pathogenesis, and industry. Two broad types of bacterial envelopes are distinguished based on whether a species retains the Gram stain (4). Both types of envelope encase a phospholipid bilayer plasma membrane (PM, or inner membrane [IM]). In Gram-positive bacteria, the PM is surrounded by a thick layer of peptidoglycan (PG), into which in some cases, teichoic acids are interwoven. In Gram-negative bacteria, this layer of PG is much thinner and is itself surrounded by a second lipid bilayer, called the outer membrane (OM), consisting of phospholipids only on the inner leaflet and lipopolysaccharides (LPS) on the outer leaflet. Both Gram-positive and -negative envelopes can show a final outer layer made up of a single protein called the S layer or a capsule of polysaccharides. Exceptions exist such as the *Corynebacterineae* suborder, including Mycobacterium tuberculosis, where the cells only moderately retain the Gram stain, due to the presence of a relatively thin PG layer surrounded by an arabinogalactan layer to which mycolic acids are covalently bound (5).

The thick, mechanically strong PG layer of Gram-positive bacteria provides the cells with an exoskeleton allowing them to survive in dilute liquid environments where a high intracellular turgor pressure is unavoidable (6). In Gram-negative bacteria, many antibiotics and other antimicrobials encounter the significant permeation barrier of the OM, prohibiting them from reaching their periplasmic or cytosolic targets (7). Various surface-exposed molecules, such as adhesins, S-layer proteins, LPS, or capsular polysaccharides, offer a means for bacterial symbionts and pathogens to interact with their eukaryotic hosts in antigen presentation and receptor-mediated adhesion (8). Pili and flagella participate in adhesion, mechanotransduction, transfer of genetic material during conjugation, and bacterial motility (9–13). Finally, integral membrane proteins traversing the PM or OM take part in many crucial functions, such as nutrient uptake, antibiotic extrusion, ATP synthesis, stress transduction, conjugation, and protein secretion (14). Accordingly, the cell envelope is highly organized and heterogeneous, therefore requiring high-resolution probing techniques to understand its structure in relation to function.

## USING FORCE TO LOOK AT THE CELL ENVELOPE

Thorough ultrastructural investigation of bacterial envelopes has historically relied on scanning and transmission electron microscopy (EM) techniques, which have the necessary resolution to allow, for example, observation of the different layers of Gram-positive and -negative envelopes as well as the crystalline arrangement of S-layer proteins (15, 16). Cryo-EM techniques have allowed solving the three-dimensional structures of membrane-spanning proteins and complexes at resolutions of a few angstroms (17). EM techniques, however, have several limitations, including often harsh chemical procedures in sample preparation and the necessity to work under vacuum and/or cryogenic conditions, precluding the possibility of investigating samples under physiological conditions.

**FIG 1:**
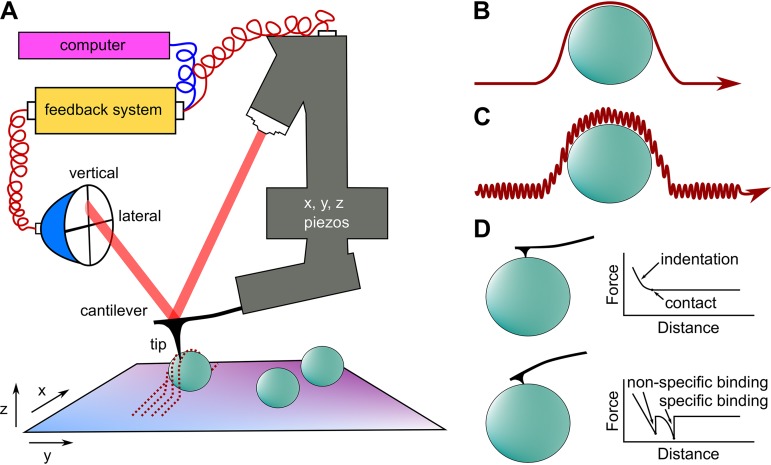
AFM as a tool to investigate the bacterial cell envelope. (A) A laser beam is focused on the end of a soft cantilever onto which a tiny probe (“tip”) is attached. The cantilever displacement is controlled by *x-y* and *z* piezoelectric elements, enabling precise movement of the tip across the cellular sample. The force exerted on the tip causes deflection of the cantilever, which is registered by a photodiode. (B) Contact mode imaging involves moving the tip laterally across the sample in a raster scan fashion and recording height data from the piezo displacement. (C) Tapping mode imaging is based on oscillating the cantilever near its resonance frequency, enabling limitation of sample damage to a greater degree than in contact mode. (D) In force-distance spectroscopy, the tip is brought into contact and subsequently retracted from the sample. The vertical deflection of the cantilever is expressed in units of force (piconewtons or nanonewtons) and plotted as a function of *z*-piezo displacement (nanometers). From the approach portion of the curve (top), the sample stiffness of the bacterial sample can be derived. From the retraction part, one can obtain the binding strength between the tip (e.g., functionalized with hydrophobic chemical groups or bioligands) and the sample (nonspecific hydrophobic interactions or specific ligand binding forces).

Within the past 30 years, atomic force microscopy (AFM) has revolutionized the way microbiologists explore the bacterial cell envelope. Not only does AFM bring a resolution that can compete with EM, but it is able to work with living (or native) samples in a natural aqueous state at physiological temperature (18–20). The principle of AFM relies on a micrometer-sized probe terminated with a very sharp tip attached to a cantilever. The cantilever is connected to a set of piezoelectric elements controlling its *x*, *y*, and *z* movement (Fig. 1A). In its basic contact imaging mode, the tip is moved laterally across the sample (Fig. 1B), while a laser focused on the cantilever is reflected onto a photodiode that can detect vertical displacements and torsional deformation of the cantilever as it comes into contact with and moves across the relief of the sample (Fig. 1A). A feedback system ensures that the force exerted by the tip on the sample does not exceed an arbitrarily small maximum, thereby protecting for example a delicate bacterial sample from damage during imaging. Provided that the deflection sensitivity and nominal spring constant of the cantilever are known, the exact force exerted by the AFM tip on the sample can be assessed from the displacement of the cantilever registered by the photodiode. Data collected from the feedback mechanism itself can provide useful contrast in imaging. These include torsional deflection (sensitive to frictional forces), vertical deflection, and the error signal obtained at each cycle of the feedback loop, all of which are sensitive to sharp changes in the surface relief. In tapping mode, the AFM tip oscillates while the sample is scanned and makes only intermittent contact with the sample, thus limiting tip-sample forces and minimizing sample damage (21–23) (Fig. 1C). In recent years, high-speed (HS) AFM modalities have been developed, enabling the tracking of single bacterial proteins and cells at high temporal resolution (24). Recording force-distance (FD) curves (19, 25) (Fig. 1D) enables the quantification of cell wall mechanics (6, 26, 27) and the determination of the localization and binding strength of single proteins (28, 29). Modern multiparametric AFM technologies allow one to record FD data across large arrays at high resolution and speed, so that maps detailing the ultrastructure, adhesion, and mechanical properties can be ascertained at the nanoscale on bacterial cell surfaces (27). Here, we review recent AFM studies that have improved our understanding of the architecture of the bacterial envelope, at (nearly) molecular resolution, under conditions not accessible by standard microscopy. For a survey of earlier work or more details on the technique, see, e.g., the reviews in references 30 and 31.

## VISUALIZING THE DYNAMICS OF SINGLE MEMBRANE PROTEINS

HS-AFM has gained great interest in the visualization and structural characterization of biomolecules, such as membrane proteins, when these are appropriately prepared on an atomically flat and regular surface (23, 24, 32–35). Here, the true power of AFM to resolve biological structures down to subnanometer spatial and ∼0.1-s time scales really becomes evident (36). A great advantage over cryo-EM and X-ray crystallography techniques is that samples can be assayed in native-like lipid membranes, in aqueous buffer, and at pressures and temperatures permissive for optimal enzymatic functions (for a comprehensive review on the topic, please refer to reference 31). These benefits of AFM allowed, for instance, subnanometer-resolution characterization of purple membrane surface structures (37), and HS-AFM, in particular, allowed the real-time visualization of cyclical conformational changes in the F_1_ ATPase rotary motor protein, revealing a structural basis for a unidirectional rotary motion during ATP hydrolysis (38).

Recently, Ruan et al. used HS-AFM to study the aspartate-sodium symporter of the archaebacterium Pyrococcus horikoshii as this integral membrane protein underwent dynamic structural changes while performing its transport function (39). They observed the protomers of the trimeric protein with dimensions of approximately 2 nm in diameter and height and separated from each other by 5.5 nm. Each protomer transport domain was observed in either an outward- or inward-facing conformation, which changed dynamically in 1.8-nm up-and-down elevator motions with dwell times of 2 to 7 s. Observing these motions allowed the authors to delineate a dynamic basis for substrate transport relying on substrate concentrations. The same group also characterized conformational changes in the pH-sensitive Gloeobacter violaceus ligand-gated ion channel (GLIC) under active buffer exchange (40). By changing the buffer pH from 3.4 to 7.5, they witnessed stunning dynamics in the GLIC conformations with the molecules wiggling around their positions and the extracellular domains tightening in on their central cavities before the channels stabilized, while changing the pH back to 3.4 reversed these conformational changes. Uchihashi et al. explored ClpB, an integral membrane protein that helps to refold toxic protein aggregates in an ATP-dependent mechanism (41). They observed dramatic conformational changes in ClpB hexamers upon binding to and hydrolysis of ATP, while mutants showed altered oligomeric forms and structural dynamics, providing new insights into ClpB-mediated disentanglement of incorrectly folded proteins.

So far, HS-AFM technologies have found limited use in the study of living samples (42), whose analysis is impeded by their large size and curvature (43). Nevertheless, Yamashita and colleagues were able to observe holes on top of living Magnetospirillum magneticum resembling OM proteins, such as porins (42), and more recently, Kumar et al. developed a new HS-AFM protocol using high-resonance frequency; small, soft cantilevers; optimized scanning parameters; and an optimized imaging buffer allowing visualization of single proteins in curved membranes (44). They used this technique to visualize single Rhodobacter sphaeroides photosystem-protein complexes within spherical chromatophore vesicles, revealing complete high-resolution images of protein complex organization within the vesicles. This is an incremental step toward the resolution of tertiary or quaternary structures of proteins by AFM on the surfaces of living cells under physiologically relevant conditions.

Force-distance curve multiparametric modalities are also starting to show promise to gain structural and functional insights on single integral membrane proteins at high spatiotemporal resolution. Focusing on integral outer membrane proteins, Mulvihill et al. used malto-oligosaccharide-functionalized tips to visualize single trimers of the LamB maltoporin and simultaneously measure binding forces and frequencies of single LamB pores and their malto-oligosaccharide ligands (45). High-resolution topographs allowed distinction of LamB trimers exposing their extracellular side from ones exposing their periplasmic side, based on ∼3.3- and ∼1.7-nm protrusions extending from the liposomal surface, respectively. A great advantage of FD curve-based AFM is that by varying the retraction speed of the probe, the free-energy landscape of a protein-ligand interaction can be approximated from the dynamic force spectroscopy data. The authors could in this way calculate binding equilibrium free energies and, from those, dissociation constants for maltotriose, maltotetraose, and maltopentaose of 8.8 mM, 2.3 mM, and 1.8 mM, respectively. Finally, while it was previously posited that binding of malto-oligosaccharides to LamB could occur in either a symmetric or an asymmetric fashion, the authors demonstrate for the first time that binding occurs preferentially on the periplasmic side and is therefore asymmetric.

## THE MOLECULAR ORGANIZATION OF THE CELL WALL: INSIGHTS FROM PURIFIED SACCULI

PG plays a central role in controlling cell shape, growth, and division. In recent years, AFM has been increasingly important in revealing the complex architecture of PG and providing insights into how it is synthesized and remodeled. In living cells, PG is closely associated with a range of other molecules and in many cases (including all Gram-negative bacteria) is hidden in live cells from direct AFM imaging by other components of the cell envelope, such as the outer membrane, making direct analysis difficult. Thus, studies of extracted cell walls (sacculi) provide essential information on underlying PG architecture (46).

**FIG 2:**
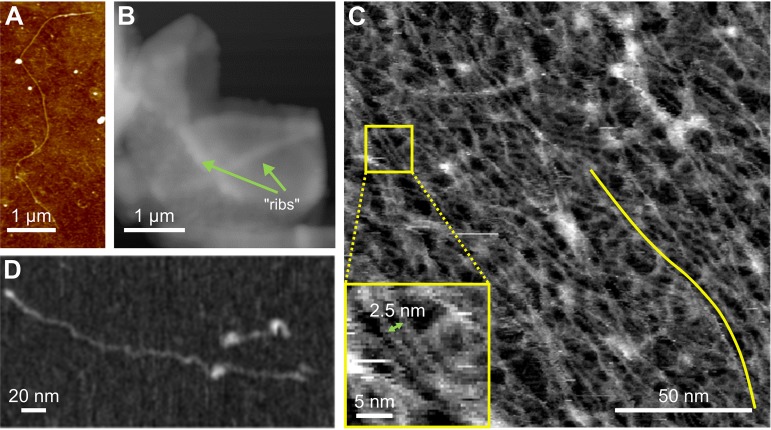
AFM of extracted peptidoglycan. (A) Topographic images showing a glycan chain from Bacillus subtilis, revealing that extremely long chains exist within the cell wall. (Adapted from reference 48 with permission of the publisher [Copyright 2008 National Academy of Sciences].) (B) Topographic image showing a peptidoglycan sacculus from S. aureus, in which orthogonal “ribs” coming from previous cell division events can be seen. (Adapted from reference 51 with permission of the publisher.) (C) High-resolution topographic image of an E. coli sacculus showing the net-like molecular organization. The yellow line indicates a single, long, glycan molecule. The inset shows parallel glycan chains with minimal separation. (Adapted from reference 52 with permission of the publisher.) (D) A glycan molecule extracted from E. coli sacculi, indicating that the cell wall contains a fraction of high-molar-mass glycan chains. (Adapted from reference 52 with permission of the publisher.)

Following pioneering work on the mechanics of sacculi extracted from Gram-negative bacteria (47), a study of the Gram-positive bacterium Bacillus subtilis (48) highlighted one of the strengths of this approach, as both the internal (PM-facing) and external surfaces of the PG could be imaged with resolution down to the nanometer scale. This revealed a surprisingly complex cell wall architecture with ∼50-nm-wide “cables” running circumferentially around the internal cell rod and a pattern of concentric or spiraling rings seen during septum formation. The same work showed that B. subtilis contained some surprisingly high-molecular-weight glycan chains, and direct imaging with AFM (Fig. 2A) revealed some of these to be multiple micrometers in length, far longer than the thickness of the cell wall and placing hard constraints on chain organization within the wall. Subsequent studies on the external surface of live ovococci also observed PG bands (49), here 25 nm wide, an observation supported by chemical mapping. Observations on ovococcus sacculi (50) found PG strands preferentially oriented around the sacculi but without “cabling.” Interestingly, features that are observed are always close to annular growth features, implying that material is rapidly remodeled after synthesis.

Application of the same AFM approach to imaging extracted sacculi from the human pathogen Staphylococcus aureus (51) was able to provide a unique insight into the unusual division mechanism in this almost spherical bacterium. As implied by its name, bacterial colonies adopt a “bunch of grapes” morphology, and this is the result of division occurring in mutually orthogonal planes, so that each division plane is at right angles to both of the previous two planes. The question arises as to how the cell “knows” in which plane to divide. AFM imaging of sacculi reveals that prior to septation the cell lays down a thick ring of PG on the internal cell wall surface (a “piecrust”) and that the remnants of these rings (“ribs”) remain across multiple division cycles (Fig. 2B), providing a potential epigenetic inheritance of where previous division planes occurred and hence a route toward determining subsequent division.

Gram-negative bacteria, in which the PG layer is sandwiched between the inner and outer membrane and is known to be only a few nanometers thick, have provided the most detailed data to date. Recent work on Escherichia coli (52), building on previous combined AFM/superresolution optical studies (53), has provided true molecular-resolution images of extracted, intact sacculi (Fig. 2C). Individual chains with a width of 1.4 nm are resolved, some of which are over 100 nm in length, in itself a surprising observation confirmed by size-exclusion chromatography and direct imaging with AFM of individual, extracted glycan molecules (Fig. 2D). The minimum separation between parallel chains in the sacculi is found to average 2.7 nm, indicative of the potential length of the relaxed peptide cross-links. The sacculus itself consists of a fine meshwork which is disordered (i.e., noncrystalline) but has a mean orientation around the cell’s circumference. Interestingly, this orientation is preserved in the cell poles, implying that it is driven by the requirements of the synthesis machinery rather than by the need to align glycan chains with the direction of principal stress in the cell wall, as although this is circumferential in the cell rod, the stress is isotropic in the hemispherical poles. Work on mutants lacking MreB, which forms multiprotein filaments guiding PG synthesis, or in which MreB has been inhibited with A22, finds that this orientational order is lost and that this correlates with the loss of the rod shape. At the same time, long chains are no longer observed, implying that it is MreB-associated synthesis that results in high-molecular-weight glycan chains. The Gram-negative cell wall is a unique two-dimensional (2-D) structural biomaterial, which can now be imaged with submolecular resolution, providing a fascinating system to explore the relationship between chemistry, mechanics, and function in a living system.

## UNRAVELING THE ULTRASTRUCTURE AND DYNAMICS OF WHOLE CELLS

**FIG 3:**
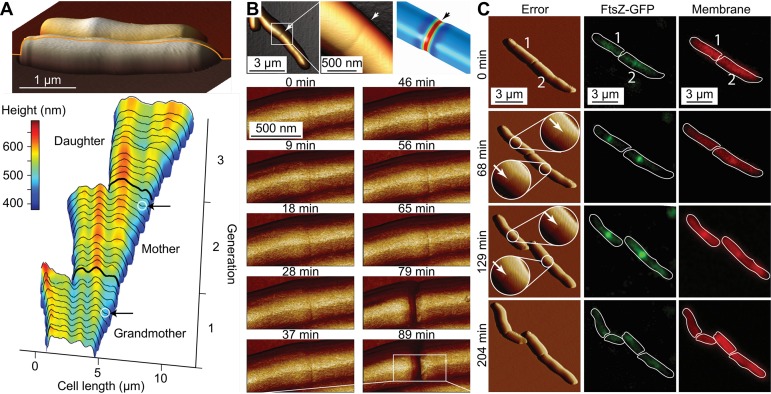
Time-lapse AFM imaging of bacterial cell growth and division. (A) Height profiles from time-lapse 3-D AFM data tracking one cell through 3 generations. Cell division occurs within wave troughs that the mother cell inherited from the grandmother cell to form daughter cells. (Adapted from reference 57 with permission of the publisher.) (B) Mycobacterium smegmatis forms a precleavage furrow (small depression marked by arrows) at the site of future cell cleavage. (Adapted from reference 59 with permission of the publisher.) During the division cycle, stress is accumulated at the precleavage furrow, leading to an abrupt fracture-like cell separation. (C) The precleavage furrow is colocalized with FtsZ ring formation and septum synthesis. GFP, green fluorescent protein. (Adapted from reference 59 with permission of the publisher.)

One of the most powerful attributes of AFM when it comes to the study of bacteria is its ability to image living cells in physiologically relevant media. During processes such as cell growth, cell division, or cell death, major changes to the outside of the cell can be observed by AFM with nanometer resolution in a time-resolved manner. Using the ability to image the same area on live cells over a period of 1 h, Touhami et al. were able to follow cell splitting of Staphylococcus aureus (54). Wu et al. studied *Mycobacterium* sp. using time-lapse AFM for several hours, during which they observed the changes in cell stiffness upon treatment of the cells with ethambutol (EMB) (55, 56). They found that the sensitivity of *Mycobacterium* sp. strain JLS to EMB is dependent on the division phase of the individual bacterium and that the equivalent spring constant of the bacterial cell degrades in conjunction with a visual degradation observed by AFM contact mode measurements. Extending the duration of AFM time-lapse microscopy experiments beyond a few hours causes technical difficulties. As cells grow and metabolize medium, they also excrete various substances that can be picked up by the AFM tip and alter the tip-sample interaction. In traditional AFM modes such as contact mode and tapping mode, such changes in the tip-sample interaction cause the AFM to become unstable and require operator interference in order to continue the measurements. In addition, depletion of nutrients and evaporation of water require constant attention by the operator, making time-lapse experiments over many bacterial division cycles cumbersome. Eskandarian et al. have overcome this problem by using the imaging mode PeakForce tapping to achieve time-lapse imaging of up to 1 week (57). By tracking a single bacterium over multiple generations, they found a correlation between morphological features visible on the surface of the bacteria (wave troughs) and future sites of cell division (Fig. 3A). These wave troughs are formed already in the previous generation and are inherited by the daughter cells to mark their future site of division. That makes the formation of wave troughs currently the earliest known event in division site selection in rod-shaped bacteria. In a later paper from the same group, Odermatt et al. used a custom time-lapse AFM in conjunction with fluorescence microscopy (58) to track the events leading to *Mycobacterium* cell division at high resolution. Using the stiffness information from PeakForce quantitative nanomechanical mapping (QNM) measurements, they found that mechanical stress concentrates around a cell in a small ring of ca. 50 nm in width (Fig. 3B) (59). This stress concentration is colocated with FtsZ-ring formation and the future division site (Fig. 3C). They postulated that this stress concentration is driven by the bacterial turgor pressure. Once the level of stress exceeds the mechanical tensile strength of the cell wall, the cells abruptly cleave in a ductile fracture mode. To prove this hypothesis, they showed that adding additional stress with the AFM cantilever tip at the future division site could trigger near-instantaneous cell separation. Conversely, increasing the cell wall strength by reducing hydrolase activity leads to cells that are incapable of separation and instead form long chains of unseparated cells. Remarkably, these cells could nevertheless be induced to separate by applying large external forces with the AFM cantilever. This example demonstrates the possibilities when AFM is used not only for measuring topography but also for measuring mechanical properties, or even for cell manipulation.

Multiparametric imaging has also recently offered exciting new possibilities to simultaneously map the structure, adhesion, and mechanics of cell envelopes in living bacteria. Imaging of Staphylococcus aureus revealed that zinc induces a major remodeling of the cell surface, i.e., the surface becomes smoother, stiffer, and stickier in the presence of this cation, a phenomenon that activates cell-cell adhesion during biofilm formation (60). Multiparametric imaging also showed that some S. aureus bacteria are surrounded by the cationic polysaccharide intercellular adhesin (PIA), which forms a soft and adhesive matrix of extracellular polymers (61).

## REAL-TIME EFFECTS OF ANTIMICROBIALS ON THE CELL ENVELOPE

**FIG 4:**
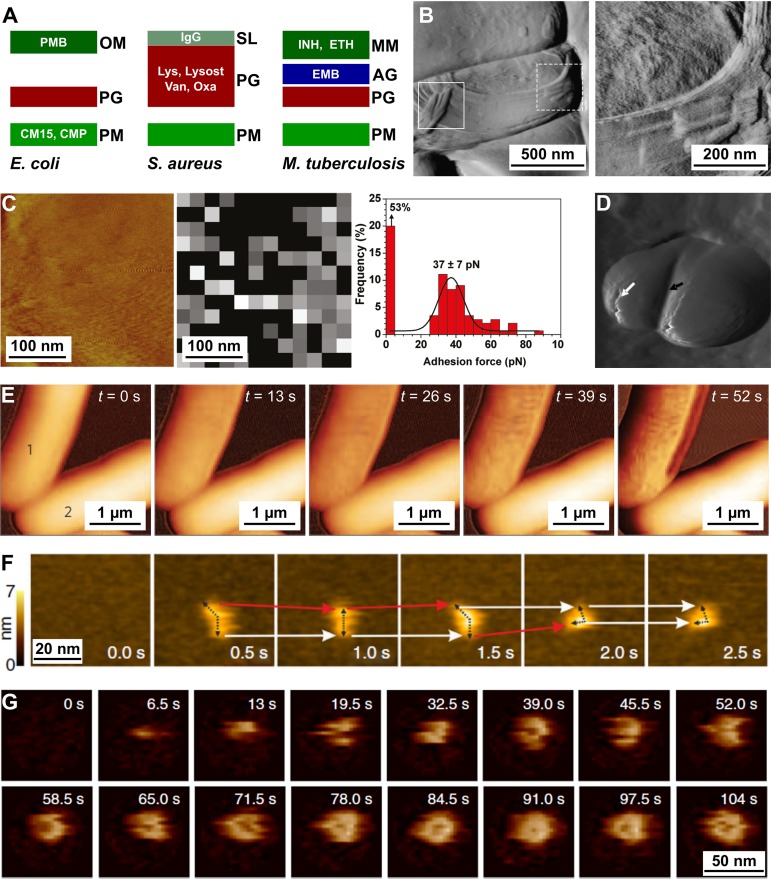
AFM has helped unravel how antimicrobials impact the cell envelopes of bacteria. (A) Schematic of the effects of antimicrobials on the cell envelopes of E. coli, S. aureus, and M. tuberculosis. PM, plasma membrane; PG, peptidoglycan; OM, outer membrane; SL, S-layer; AG, arabinogalactan; MM, mycomembrane; PMB, polymyxin B; CM15, antimicrobial peptide; CMP, complement; IgG, immunoglobulin G; Lys, lysozyme; Lysost, lysostaphin; Van, vancomycin; Oxa, oxacillin; INH, isoniazid; ETH, ethionamide; EMB, ethambutol. (B) Contact mode deflection images of M. bovis BCG treated with EMB. Squares indicate pronounced cell surface alterations. In the right panel, an enlargement of the dashed box in the left panel is shown. (Adapted from reference 62 with permission of the publisher.) (C) Detection of lipoarabinomannan (LAM) on isoniazid-treated M. bovis BCG cells with probes functionalized with antilipoarabinomannan antibodies. The left panel shows a high-resolution contact mode height image, and the middle panel shows an adhesion map recorded on a bacterium. The right panel shows the corresponding adhesion force histograms. (Adapted from reference 64 with permission of the publisher.) (D) Nanoscale perforations on lysostaphin-treated S. aureus cells. The black and white arrows point to the splitting septum and perforations in the cell wall, respectively. (Republished from reference 6.) (E) Alterations in surface ultrastructure occurring in an E. coli cell (cell 1) within 1 min of treatment with CM15. The alterations are initially resisted in cell 2, which started to show alterations after 78 s (not shown). (Adapted from reference 65 with permission of the publisher.) (F) HS AFM time series of antibody movement on its antigenic surface. Red arrows indicate Fab arms undergoing movement, while white arrows show Fab arms that remain stationary. (Adapted from reference 68 with permission of the publisher.) (G) Oligomerization of the MAC in model membranes. (Adapted from reference 70 with permission of the publisher.)

Several important antibiotics, such as β-lactams, glycopeptides, and antitubercular drugs, target the synthesis of components of bacterial cell envelopes. In addition, envelopes are also vulnerable to the effects of cell wall-hydrolyzing enzymes like lysozyme and molecules produced as part of the immune response, including antimicrobial peptides, antibodies, and complement. The high resolution and sensitivity of AFM have brought novel insights into the real-time effects of antibiotics and external agents on the cell envelope ultrastructure (Fig. 4A).

Nanoscale cell wall surface alterations in live Mycobacterium bovis BCG cells treated with EMB, which inhibits synthesis of the arabinogalactan polysaccharidic layer that encapsulates the PG layer in mycobacteria, were first observed by Verbelen et al. (62) (Fig. 4B). Ethambutol treatment at a concentration close to the MIC leads to significant nanoscale alterations in the normally smooth surface topography of the mycobacterial cell surfaces, with the apparent partial loss of layers of the mycomembrane and appearance of concentric rings possibly reflecting the highly elusive arrangement of intact arabinogalactan attached to PG. Similarly, treatment of mycobacterial cells with isoniazid or ethionamide, antibiotics that target the synthesis of mycolic acids in the cytosol of the bacteria, leads to alterations in the normally smooth surface topology of these cells (63). Finally, FD-based AFM unraveled the localization of lipoarabinomannan in deeper layers of the mycobacterial cell envelope when mycobacteria were first treated with isoniazid (64) (Fig. 4C). Time-lapse AFM has allowed observation of the real-time degradation of the S. aureus cell wall by lysostaphin, an enzyme that targets PG (6). Notably, the high resolution of AFM contact mode topographic images revealed swelling of the cells, nanoscale perforations, and septal splitting and was sensitive to increases in surface roughness in the lysostaphin-treated cells (Fig. 4D).

The effect of some drugs on the cell surface ultrastructure may occur within milliseconds, which cannot be captured with conventional AFM techniques. Fantner et al. developed special AFM cantilevers allowing high-speed tapping mode AFM and observed the activity of an antimicrobial peptide (CM15) degrading E. coli cells at high resolution (1,024 by 256 pixels) in a time frame of a few seconds (65) (Fig. 4E). The technique was further refined by inducing actuation of the cantilever by shining a second laser on it, photothermally heating it up in a highly controlled periodic way and thus sidestepping the limitations inherent to the *z*-piezo scanner (66). This technique, called photothermal off-resonance tapping (PORT), could track E. coli disruption by CM15 and B. subtilis cell wall hydrolysis by lysozyme (67).

Owing to their extremely small size, the nanoscale visuospatial and mechanical details in the binding of antibodies to the epitopes exposed by their antigens on bacterial surfaces have been elusive subjects. Real-time HS-AFM, acquiring images on the 100-ms time scale, has been used to characterize the movement of IgGs on bacterial S-layer proteins of Lysinibacillus sphaericus (68) (Fig. 4F). The movement is due to steric strain between the two paratopes on the bivalent antibody and their epitopes on the S-layer surface, resulting in an elegant model that explains how suboptimal spacing and orientation between epitope pairs drive IgG to “walk” stochastically on antigenic surfaces. Notably, higher antibody concentrations resulted in an ensemble effect where aggregates of the antibodies formed at the approximate size required for binding of the C1q component of complement (69), a step which precedes phagocytic clearance of Gram-negative pathogens. The final steps of the complement pathway involve formation of the membrane attack complex (MAC) consisting of the complement proteins C5b to C9. The fully oligomerized MAC essentially pierces holes in bacterial plasma membranes, leading to their death and lysis. Rapid real-time AFM revealed that after self-hetero-oligomerization of the C5b to C8 subunits of MAC in supported E. coli lipid bilayers, initial C9 insertion acts as the kinetic rate-limiting step of complete pore formation (70) (Fig. 4G).

Finally, studying how bacteria respond to mechanical cues is a growing field in contemporary biophysics, microbiology, and biomedical sciences (26). FD-curve measurements have demonstrated that the NsaS-NsaR two-component system in S. aureus allows the bacteria to sense the cell wall deformation stress caused by adhesion to surfaces, resulting in the upregulation in transcription of the NsaAB efflux pump gene (among others), which correlates with nisin tolerance by S. aureus biofilms (71). In a follow-up study by the same group (72), it was observed that PG-active antibiotics increased cell wall deformation of wild-type cells as well as cells lacking an enzyme involved in PG cross-linking (72).

## PERSPECTIVES

The bacterial cell envelope is made of sophisticated multicomponent structures and machineries that allow it to fulfill its essential cellular functions. Elucidating bacterial envelope structure and function requires *in situ* nanoscale analysis, where AFM has begun to enable the study of the organization, dynamics, and interactions of the envelope, either purified or in living cells, with unprecedented resolution. This has revealed complexities of cell architecture hitherto unexpected and gives us a glimpse of future possibilities in unraveling how bacteria maintain viability, grow, divide, and interact with their environment and how interventions, such as antibiotics, undermine these elegant processes.

What are the challenges ahead? AFM is inherently a surface technique, and further insights will be gained by the utilization of correlative approaches to give a broader interrogation of cell envelopes. An important direction for the next decade will be to combine AFM with high-resolution optical microscopy techniques, to image specific cell surface components and structures while quantifying their properties and interactions. In one such study, a single-molecule localization optical microscope combined with AFM was shown to enable the localization of specifically labeled proteins within high-resolution AFM images under aqueous conditions (58). AFM captured the surface structure of E. coli expressing a specific fusion protein, and superresolution microscopy provided information on the expression level and spatial distribution of the protein. We believe that it is by the development of such correlative approaches that future advances will begin to reveal the marvels of bioengineering that underpin bacterial life.

Currently, the low temporal resolution of AFM remains an important technical bottleneck for analyzing the dynamic organization and assembly of the cell envelope. Recording topographic images by classical AFM takes around 30 to 60 s, which is much slower than optical microscopy imaging. As discussed above (24), HS imaging has enabled researchers to reach millisecond resolution over the past decade, including on bacterial specimens. It is also possible to increase time resolution using near-field scanning optical microscopy (NSOM), in which an optical fiber with a nanoscale aperture captures dynamic processes on labeled membranes with nanoscale spatial resolution (73). Combined, e.g., with fluorescence correlation spectroscopy, NSOM can record dynamic events in living cells with submillisecond time resolution.

Last, correlating structural information with biophysical properties and molecular interactions is key to fully understanding the functions of the cell envelope. Multiparametric AFM imaging (27) has offered unprecedented opportunities to simultaneously image the structure, elasticity, and interactions of biological specimens, including living bacterial cells, with high spatiotemporal resolution. Some key examples are the mechanical and chemical properties of single proteins in purple membranes, mapping and probing single sensors on yeast cells, imaging the extrusion of single filamentous bacteriophages on living bacteria, and unraveling the adhesive properties of biofilm-forming microbial pathogens. We are confident that further developments of the above advanced techniques will enable microbiologists to understand the structures, dynamics, and functions of cell envelopes, down to single-molecule resolution.
